# Intraprocedural guidance for recanalization of post-thrombotic venous lesions using live overlay of center lines from pre-operative cross-sectional imaging: a preliminary experience

**DOI:** 10.1186/s42155-020-00121-6

**Published:** 2020-06-21

**Authors:** Sri Hari Sundararajan, Raphael Doustaly, Gregoire Avignon, David C. Madoff, Ronald S. Winokur

**Affiliations:** 1 Department of Radiology, Division of Interventional Radiology, New York Presbyterian Hospital/Weill Cornell Medicine, 525 East 68th Street, New York, 10065 USA; 2 GE Healthcare, 283 Rue de la Miniere, 78533 Buc, France; 3 Department of Radiology and Biomedical Imaging, Section of Interventional Radiology, Yale School of Medicine, 330 Cedar Street, TE-2, New Haven, CT 06520 USA; 4 Department of Radiology, Division of Interventional Radiology, Thomas Jefferson University Hospital, 132 South 10th Street, Philadelphia, PA 19107 USA

**Keywords:** Vessel overlay, Vessel tracking, Vessel ASSIST, Pre-procedural CT MR venography, Fluoroscopic live overlay, Chronic venous occlusion, Iliocaval thrombosis, Venous recanalization

## Abstract

**Purpose:**

Pre-procedural contrast-enhanced CT and MRI imaging is typically acquired prior to deep venous recanalization procedures for post-thrombotic syndrome. This technical note reports the utility of live-overlay of augmented centerlines extracted from pre-procedural CT and MRI imaging in facilitating fluoroscopic-guided recanalization of post-thrombotic venous lesions.

**Methods and materials:**

Six patients with pre-procedural CT or MR venography data were incorporated into a commercially available 3D overlay software (Vessel Assist, GE Healthcare, Buc, France) during venous disease interventions for post-thrombotic venous lesions. Procedures were performed on the GE Discovery IGS 740 fluoroscopy system. After manual determination of the vasculature from preprocedural CT or MR, centerlines were created representing the location and trajectory of the vessels. Steps showcasing the creation of centerlines and their representation during overlay with real-time fluoroscopic guidance in these cases are outlined. Time required to cross the post-thrombotic and occlusive venous segments were reviewed.

**Results:**

All iliocaval recanalization procedures were successfully performed utilizing vessel centerline 3D overlay. In one case where occlusion extended to the femoral vein, mis-registration was identified over the femoral anatomy due to a complex leg rotation compared to pre-procedural imaging. No procedural complications related to utilization of software were noted. Average crossing time for occlusions was 3.4 min (range 1.6–5.2).

**Conclusion:**

3D overlay with vessel tracking from pre-procedural CT and MRI imaging is technically feasible and assists in catheter navigation for post-thrombotic venous segments. While results from these preliminary experiences support the continued use of this technology, further prospective and comparative evaluation of this technique is warranted to assess for added value in technical success, reductions in procedure time or reductions in radiation exposure.

## Introduction

Recanalization of chronic deep venous obstructions or post-thrombotic venous segments can require extensive fluoroscopic time, contrast use, and repeat interventions (Barbati et al. 2019). One contributing factor to the challenge of such cases is navigating the occluded segment rather than collaterals coursing in a similar trajectory, especially when considering sharp recanalization techniques (Cohen et al. 2018). Contrast-based road mapping for device navigation can be variable in delineating occluded vein segments or patent segments leading to normal venous outflow (McDevitt et al. 2019). As such, strategies to improve operator confidence during times of catheter guidance are invaluable in achieving procedural completion and technical success. Augmented reality (AR) has been shown to improve procedure performance in other challenging areas such as neurosurgery (Contreras Lopez et al. 2019), by projecting representation of structures of interest extracted from the pre-operative exams on live imaging during the procedures. Cases using an augmented vasculature model for venous recanalization have been reported previously (Chinnadurai and Bismuth 2018). In this patient cohort, poor contrast opacification due to chronic post-thrombotic changes prevented adequate guidance for catheterization. Trajectories referred to as “centerlines” were created by using contrast-enhanced CT and MR imaging acquired prior to recanalization. These centerlines corresponding to the location of the post-thrombotic and often hypoplastic venous segment, as delineated by the interventional radiologist, can be superimposed on live fluoroscopy to improve vessel catheterization. This technical report highlights the feasibility of utilizing centerline fluoroscopy overlay from pre-operative CT and MR imaging during traversal of post-thrombotic venous lesions.

## Materials & methods

Following IRB approval, a retrospective review was performed to identify cases in which centerlines from pre-operative imaging were incorporated during venous recanalization. From July 2016 (when software was made available to the authors’ institution) to December 2017, six consecutive patients with post-thrombotic iliocaval venous lesions were identified. Low-sample size stemmed from software implementation during hours of operation in which support staff was available in the event troubleshooting was required. Five patients had pre-procedural contrast-enhanced CTs while one patient had pre-procedural contrast enhanced MRI. Patients were treated on the same angiography unit (Discovery IGS 740, GE Healthcare). Before the start of each procedure, cross-sectional images from the most recent pre-operative scans were loaded from PACS to a designated workstation (Advantage Workstation, GE Healthcare). Each dataset was prepared with one segmented volume to be used for initial registration and vessel centerline creation for the vessels of interest.

**Fig. 1:**
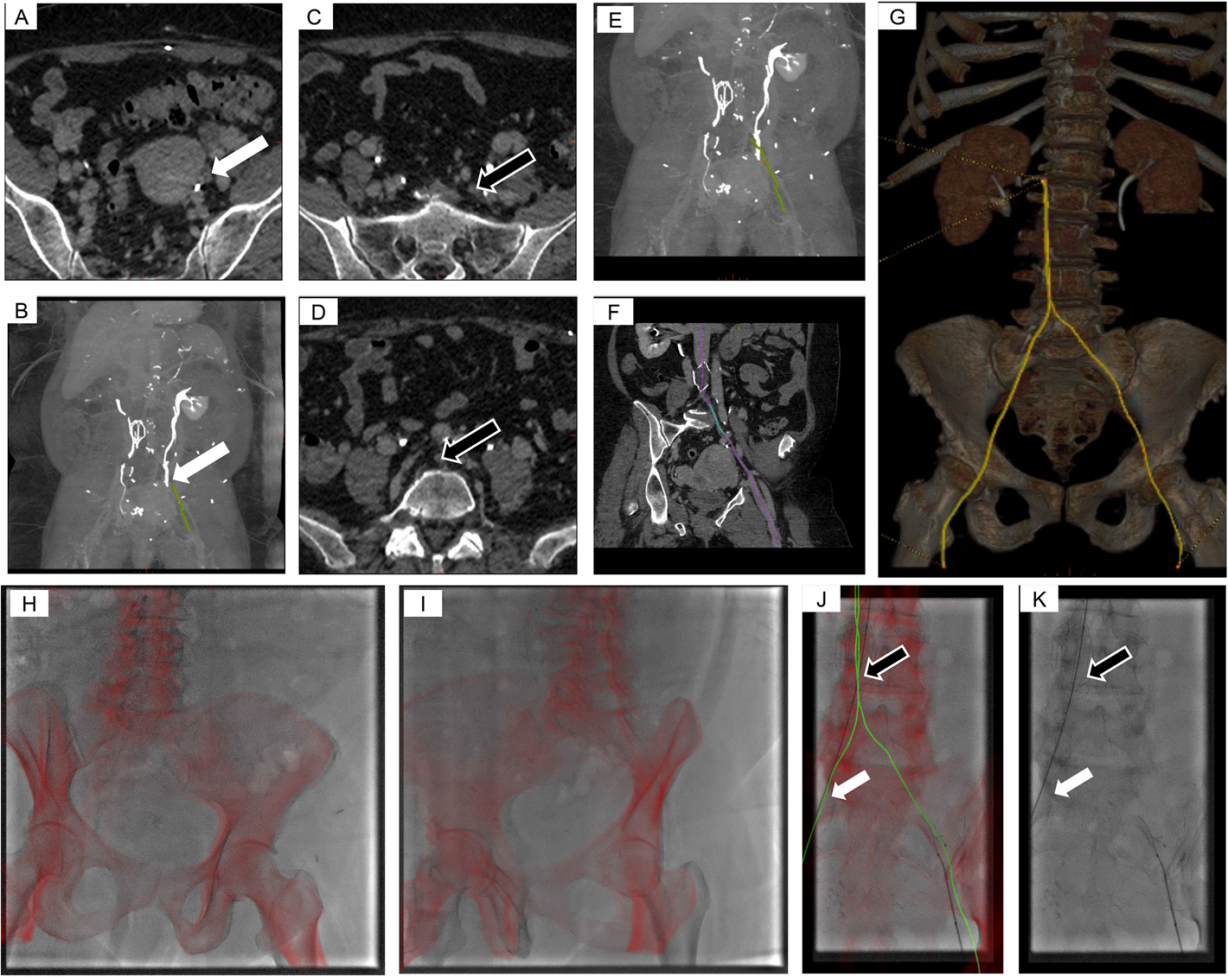
Centerline creation and roadmapping step-by-step tutorial. Begin by distinguishing bone, vessel and calcification densities from the pre-operative CT and start building the centerline for the vessels of interest using the reformatted views (**a**, white arrow on initial axial starting point). The centerline will start growing in 3D (**b**, white arrow). Once the occlusion is reached, start a bridge and deposit multiple points throughout the occluded vessel until the re-entry point (**c**-**d**) to continue growing the line (**e**). Adjust the vessel centerline using a dedicated tool (**f**). Repeat the operation on other vessels if necessary (**g**). Before proceeding with intra-procedural vessel selection, an assisting x-ray technologist fuses the pre-operative data on live fluoroscopic images using a Bi-View guided workflow all at tableside. Registration is performed by cross-referring fixed fluoroscopic landmarks (in this case, pelvic and spinal bones) with the same osseous landmarks on CT using only 2 fluoroscopic images approximately 50–60 degrees apart (in this case, RAO 15 (**h**) and LAO 40 (**i**). After initial registration, the vessel centerlines are displayed to the user (**j**), providing guidance to navigate the catheter compared to the standard fluoroscopy image (**k**). Dynamic adjustments to the registration are performed as needed by adjusting the red bony landmark overlays. Note that the occluded right common iliac vein (white arrow) and infrarenal inferior vena cava (black arrow) have already been crossed without contrast instillation using this technique

**Fig. 2:**
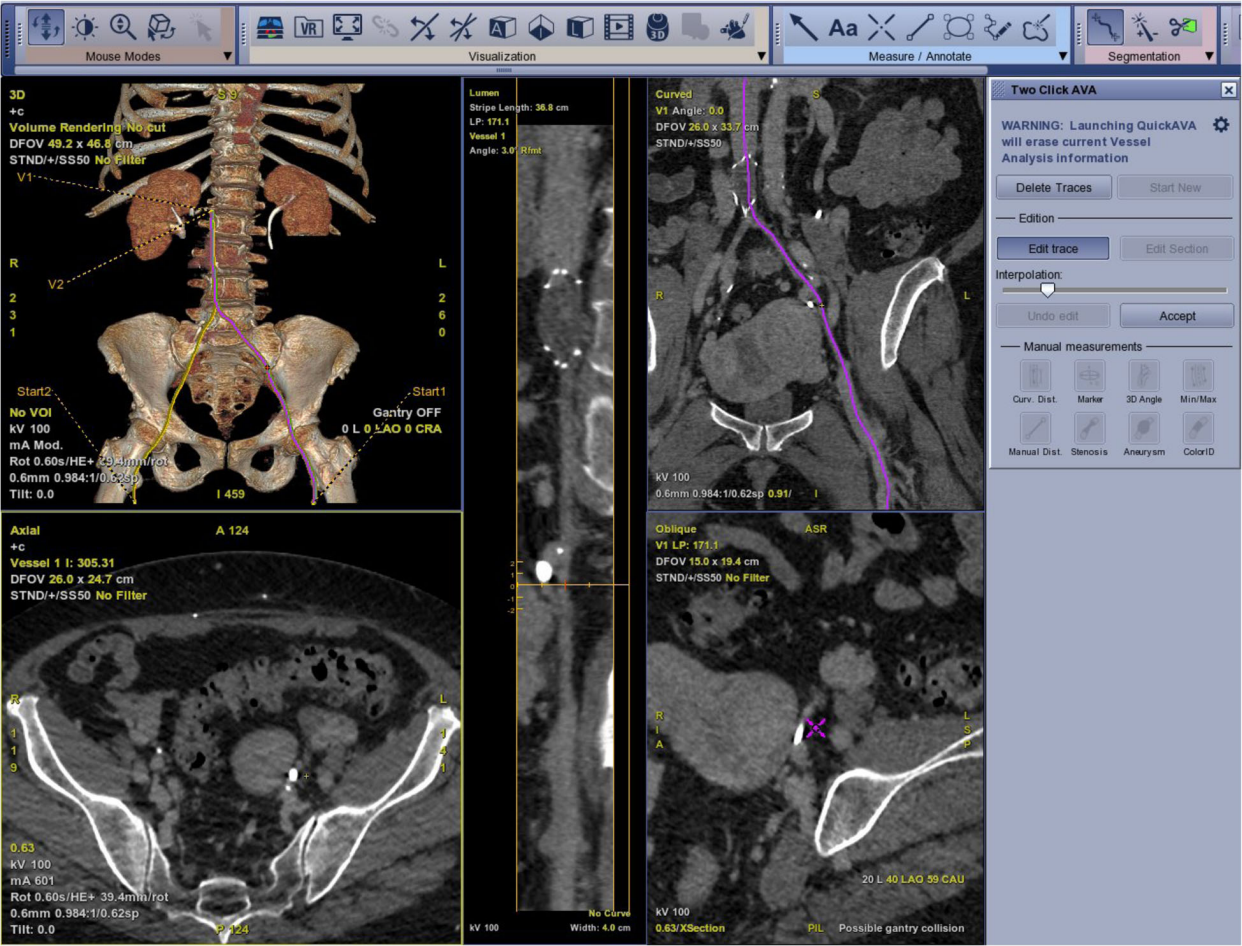
Screen grab from technologist’s monitor during center line creation from pre-procedural imaging on a patient with bilateral ilio-femoral occlusions. The centerlines are defined on each side from a point distal to the occlusion and proximal to the patent portion of the inferior vena cava

The technique for centerline creation, vessel tracking, and fluoroscopic overlay is shown in Fig. 1. To initiate the creation of the vessel centerline, multiple points of interest were manually entered in the axial projection from the CT or MR imaging. A final point was positioned to indicate the most central part of the vessel to be tracked. A preliminary template of the centerline created from those points was outlined by the software on each cross-sectional view. The user then refined the centerline curvature as needed using a dedicated tool (Fig. 1a-g and 2).

**Fig. 3:**
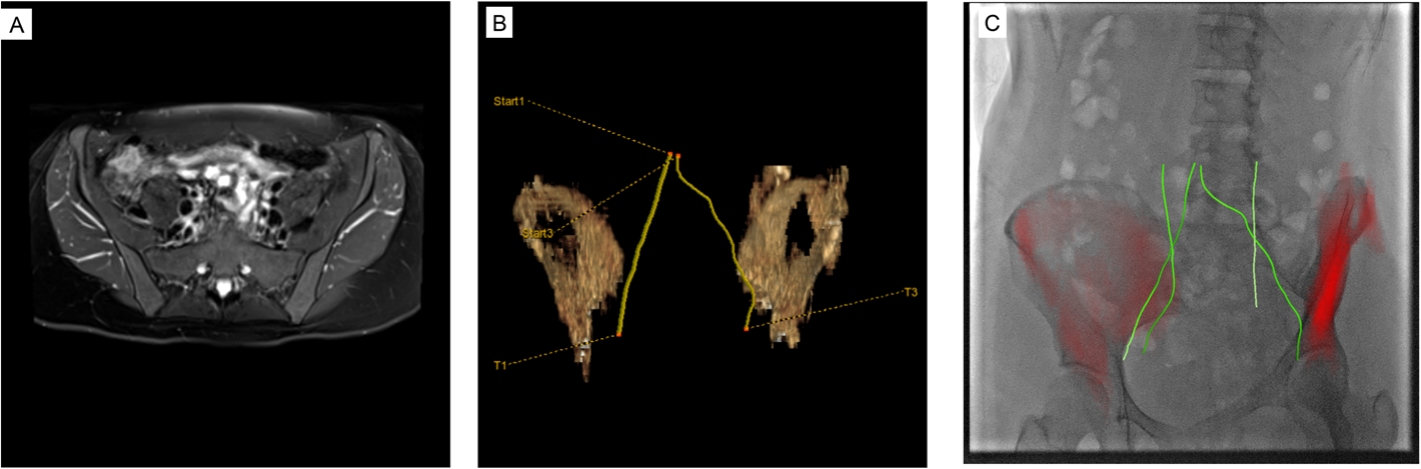
The following case is from a vascular intervention separate from the reported patient cohort (**a**). Specifically, this demonstrates the ability to use MR datasets in following vessel centerlines in a similar fashion as described in the Fig. 1 caption. The centerlines are first created (**b**). Bones are then semi-automatically segmented and registered to the fluoroscopy for further guidance (**c**)

**Fig. 4:**
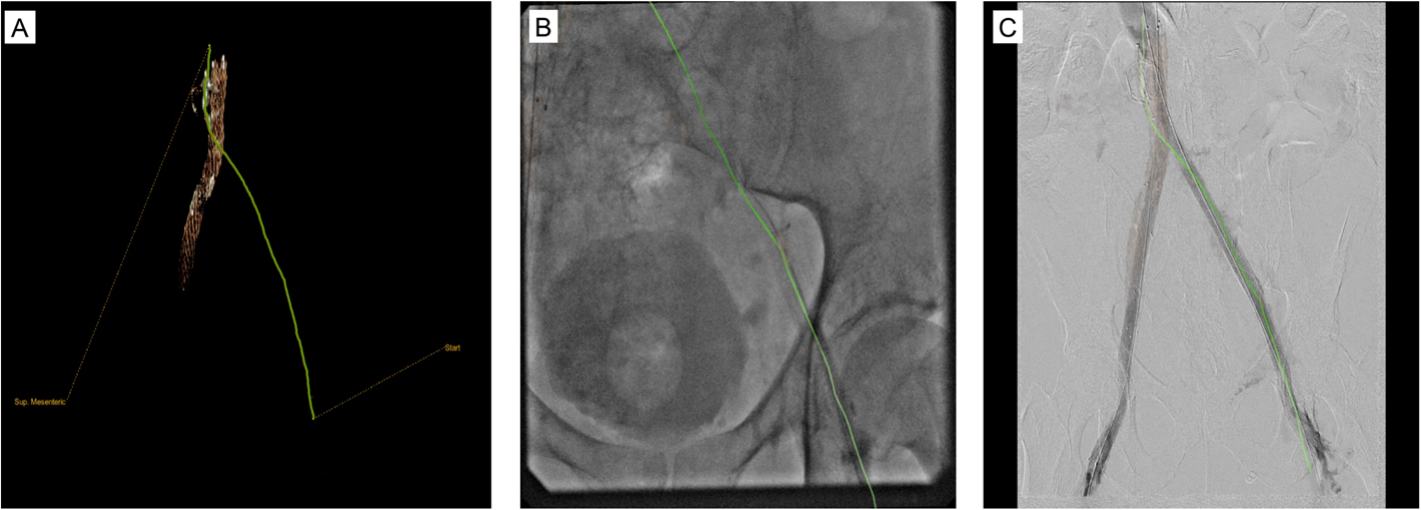
Cohort patient number 6 with a right iliac vein stent that was used for initial registration instead of the bones (**a**). The registration accuracy was considered appropriate (**b**). Final DSA under large field of view showed complete recanalization and allowed final assessment of the registration accuracy (**c**)

In cases of multivessel recanalization, the method was repeated in similar fashion on the other vessel(s). With pre-operative CT datasets, bones were used for initial registration to live fluoroscopy and were automatically segmented by the software. Pre-operative MRI datasets required manual segmentation of the femoral heads or other structure of interest to allow for registration to live fluoroscopy (Fig. 3). In patients with pre-existing stents (if present in a different vascular segment than the one requiring recanalization), initial registration could be supplemented with these fixed reference points rather than the bones alone (Fig. 4).

Once the patient was on the angiography table, and before the procedure started, the segmented anatomy and the vessel centerlines from the pre-operative scan were loaded into the overlay software and registered to the patient by the technologist using two fluoroscopic images 60^0^ apart (Fig. 1h-i). The overlay display was made available to the operators on the large display monitor, side-by-side with the native fluoroscopic images (Fig. 1j-k). Refinement of the registration was performed if the operators noticed mis-registration. Digital zoom was also used with the overlay display to reduce the need of field-of-view magnification related exposed dose. Stored fluoroscopic images of each case were retrieved and time information reviewed, to compute the occlusion crossing times.

## Results

Table 1 summarizes the six patients, their presentation pathologies, procedural parameters, and clinical follow-up. Technologists performed the overlay setup in less than 5 min in all cases. No errors or difficulty in setup were reported. The physicians for all iliocaval cases considered accuracy of the registration appropriate. In one patient, where the occlusion extended to the femoral vein, mis-registration was identified over the femoral anatomy due to differential leg rotation compared to CT. This was corrected with manual translation and rotation readjustments using osseous segmentation as a landmark. No procedural complications related to software utilization were recorded. Time to cross the post-thrombotic venous segments with a hydrophilic 0.035″ wire was on average 3.4 min, ranging from 1.6 min to 5.2 min among all cases. In one patient, no contrast was utilized to visual the post-thrombotic venous segment during intra-procedural catheterization due to the presence of the centerline guidance.

**Table 1:** Patient demographics and cohort-specific venous pathology with follow-up

#	Age	Gender	Preprocedural Imaging	Time between Imaging and Procedure	Proximal Extent	Distal Extent	Pathology	Occlusive	Change between Imaging and Procedure?	Crossing Time (minutes)	Follow-up (time and patient status)
1	55	F	CT abdomen/pelvis (90 s contrast delay)	1 month	IVC (Below Filter) Occlusion	Bilateral Femoral Vein Occlusion	Metastatic ovarian carcinoma. IVC and iliac vein thromboses related to extrinsic compression and carcinoma-related hypercoagulability.	Yes	No	4.2	1 year followup (no further visits), no recurrent occlusion, continued compression stockings
2	52	M	CT abdomen/pelvis (90 s contrast delay)	3 months	IVC (Below Filter) Occlusion	CIV Confluence Occlusion; Bilateral CIV and EIV Stenosis	RLE provoked DVT in 2015 following lumbar spinal surgery. IVC filter placed. Course complicated by progression of thrombosis and post-thrombotic syndrome.	Yes	Yes, new occlusion below iliocaval confluence	5.2	5 year followup, no recurrent ileocaval occlusion, continues daily Coumadin 2 mg for chronic scarring of bilateral femoroal-popliteal veins
3	62	M	MRI venogram abdomen/pelvis	1 month	Left CIV Stenosis, Left EIV Occlusion	Left Popliteal Occlusion	Unprovoked LLE DVT in 2016. Subsequent post-thrombotic syndrome with femoral vein stenosis and occlusive popliteal vein stenosis.	Yes	Yes, progression of thrombosis to include left EIV (initially from CFV)	3.0	2 year follow-up (no further visits), no recurrent iliac stenosis/occlusion. Persistent mid left femoral vein occlusion with post-thrombotic change, switched to Xarelto and Aspirin 81 mg daily
4	54	M	CT abdomen/pelvis (90 s contrast delay)	4 months	Right EIV to CFV Confluence In-Stent Stenosis	Distal Right CFV Stenosis	Morbidly obese patient with pulmonary hypertension, sarcoidosis, and IVC narrowing. Prior stenting from intrahepatic IVC to iliac bifurcation in 2015. Multiple subsequent venoplasties and stent extensions for lower extremity symptomatic control.	No^a^	N/A, diagnosis could not be made on CT	1.6	4 year followup, no further stenting, decreased frequency of interval venoplasties (yearly instead of every 3–6 months) for mild stenoses at junction of proximal IVC and right common femoral vein constructs, continues Coumadin 3 mg and Aspirin 81 mg daily
5	64	F	CT abdomen/pelvis (90 s contrast delay	1 month	IVC (Below Filter) Occlusion	Bilateral EIV Occlusion	Morbidly obese patient with history of breast carcinoma, positive lupus anticoagulant, and DVT/pulmonary embolism following gastric bypass in 2010 at outside hospital. IVC filter placed. Following transition of care to current institution, note made of progressed iliocaval thrombotic burden below filter with post-thrombotic syndrome.	Yes	No	3.8	4 year followup, no recurrent occlusion, continuing daily Xarelto
6	36	M	CT abdomen/pelvis (90 s contrast delay)	2 weeks	Left CIV In-Stent Occlusion	Left Femoral Vein	History of Factor V Leiden, May Thurner Syndrome, and multiple prior LLE extremity interventions. Rethrombosis of indwelling stents within the left CIV, EIV, and CFV.	Yes	No	2.4	3 year followup, recurrent occlusion 1 year following initial procedure, subsequent thrombolysis and thrombectomy with repeat occlusion 1 year following this, no further interventions given low likelihood of recanalization, continues daily Aspirin 81 mg and Fondaparinux

^a^Given body habitus, patient comes for routine venoplasty of stents depending on symptoms

## Discussion

While the benefits of fluoroscopic 3D overlay created from procedural digital subtraction angiography and cone-beam CT acquisitions are well demonstrated, the utility of incorporating peri-operatively acquired imaging data during vascular interventions remains limited (Gorges et al. 2006; Bapst et al. 2016; Tacher et al. 2017). This report showcases the successful use of 3D overlay of centerlines from pre-operative imaging as roadmaps during venous recanalization interventions in six different patients.

Contrast-enhanced CT and MR imaging was sufficient for the provider to identify the course of the post-thrombotic venous segments and allowing for creation of a centerline. The software automatically calculates trajectory based on luminal opacification of the proximal and distal ends in the contrast enhanced cross-sectional examination with registration of the non-opacified portions of the vessel performed by bridging segments with manual clicks by either the operator or technologist. Such automatic registration does not correlate well with vessel wall thickness due to poor wall visibility relative to intraluminal post-thrombotic changes limiting the software’s ability to display a theoretical outline of the vessel.

Currently, the implemented centerline technology relies on physician determination of the affected post-thrombotic venous channel. In the future, it may be possible to utilize this technology with non-contrast imaging based on landmark selection and triangulation with a selected vascular tract, though this was not formally interrogated in this study. Further automated segmentation of anatomical landmarks from imaging datasets for registration with fluoroscopic images may also be considered in the future.

The use of augmented centerlines represents a promising road mapping tool regardless of the position of the C-arm. Displaying vessel centerlines allows for guidance through long segments of poor vessel opacification of contrast due to post-thrombotic changes. In the future, an augmented reality environment could be overlaid to the patient using more advanced visualization tools such as virtual reality glasses.

Limitations of this technical note include a small retrospective patient cohort and lack of a control group showing differential time to recanalization or contrast volume use. Given the superior spatial resolution and standardization of dose parameters of CT compared to MRI, CT is preferred in the identification, extrapolation, and standardization of centerlines from occluded and atretic vascular channels over MR. However, in patients with prior MR imaging for other indications, these can be alternative options to develop guidance overlay centerlines. In one patient with pre-existing right external iliac to femoral vein stents and a left iliocaval post-thrombotic occlusion, a crossing time of 1.6 min was recorded, which is lower than others in this cohort. Presumably, the patient’s preexisting stents provided a more accurate landmark than the adjacent pelvic osseous structures when affixing 3D centerlines to the live images.

## Conclusion

In conclusion, 3D overlay with vessel tracking from pre-procedural CT and MR imaging data for catheter navigation during venous recanalization interventions is feasible. Procedure time to cross the occlusion was on average 3.4 min in this particular cohort, noting that reducing crossing times subsequently reduces procedure length in these often time-intensive procedures. Specifically, through a combination of the centerline creation and overlay process, intravascular ultrasound incorporation, and understanding of catheter and wire locations based on fluoroscopic landmarks, the potential for reductions in procedural contrast accumulation and dose exposure without compromising safety exists and would be fodder for future investigation in a prospective nature.

## Data Availability

All data reported in this manuscript is available for review if requested. All patient related data recorded for the purposes of this manuscript is stored in a secure HIPAA-compliant data storage repository.
